# Rudolf Virchow and the Recognition of Alveolar Echinococcosis, 1850s

**DOI:** 10.3201/eid1305.070216

**Published:** 2007-05

**Authors:** Dennis Tappe, Matthias Frosch

**Affiliations:** * University of Würzburg, Würzburg, Germany

**Keywords:** Rudolf Virchow, alveolar echinococcosis, Echinococcus multilocularis, pathology, infectious diseases, historical review

## Abstract

Alveolar echinococcosis, which is caused by the larval stage of the fox tapeworm *Echinococcus multilocularis*, is one of the most dangerous parasitic diseases. It is endemic in many parts of the Northern Hemisphere and an emerging health problem in the People’s Republic of China. In Europe and North America, human cases are rare, but concomitant with an increase in the population of the final host, the red fox, an increase of human infections is expected. Rudolf Virchow, the father of the concept of cellular pathology, determined in the 1850s that an *Echinococcus* sp. was the causative agent of this enigmatic emerging disease. In his famous publication in 1855, he described the clinical course of the disease, its macroscopic aspects, and histopathologic findings in detail. He also identified the disease formerly known as alveolar colloid of the liver to be an infection with the larval stage of an *Echinococcus* sp.

## Abstract 2

Virchow proved that the disease “alveolar colloid” was caused by an *Echinococcus* sp.

Rudolf Virchow (1821–1902, Figure 1), the originator of the concept of cellular pathology, also concerned himself extensively with the pathology of infectious diseases (*1*). In 1848, when he was working as a military physician at the Charité Hospital in Berlin, he distributed oppositional political pamphlets and was thereupon suspended from work. On the condition that he would no longer get involved with political activities, he was offered a position as a professor of pathology at the University of Würzburg, Lower Franconia, Germany. He stayed only 7 years in Würzburg, but it was during these years, from 1849 to 1856, that he made some of his major discoveries. Virchow initially worked in the Theatrum Anatomicum (Figure 2), a baroque but rather small pavilion in the center of the city. He shared the cite with Albert Kölliker (1817–1905), a contemporary anatomist who once called the place “a gaunt dive” (*2*).

**Figure 1:**
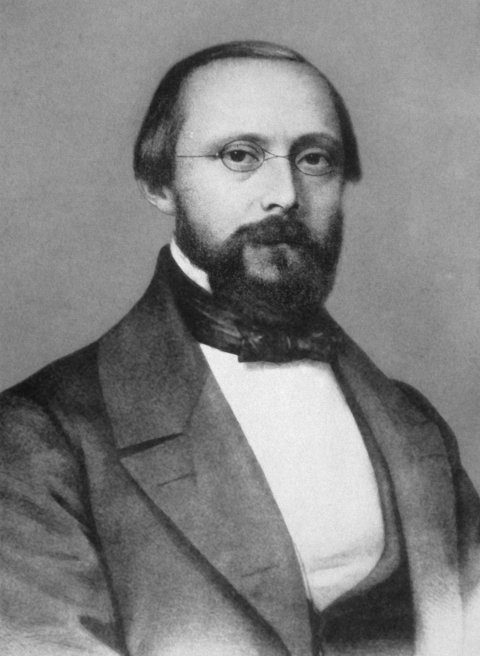
Rudolf Virchow. Photograph taken during his 7 years in Würzburg, Germany (1849–1856), as professor of pathology. Courtesy of the Institute of Pathology, University of Würzburg.

**Figure 2:**
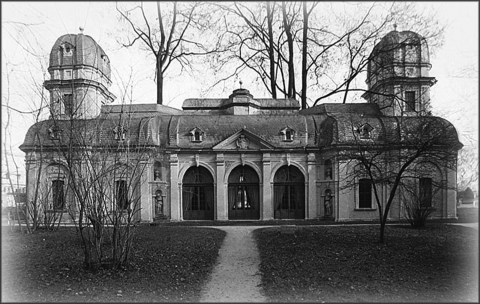
Baroque pavilion in the backyard of the Juliusspital in Würzburg. Originally a garden pavilion, it was later remodeled to form the Theatrum Anatomicum, where Virchow worked from 1849 to 1853 in the right wing. Kölliker, an anatomist, occupied the left wing. Years later, it was converted into a greenhouse. It is a conference room and exhibition hall today. Courtesy of the Institute of Pathology, University of Würzburg.

In Würzburg, Virchow soon became the secretary of the local scientific society, Physicalisch-Medicinische Gesellschaft, (Physico-Medical Society). In 1855, the society’s scientific journal Verhandlungen der Physicalisch-Medicinischen Gesellschaft (Proceedings of the Physico-Medical Society) published his observations and conclusions about a disease that we know today as alveolar echinococcosis. The title of the article was “Die multiloculäre, ulcerirende Echinokokkengeschwulst der Leber” (The multilocular, ulcerating *echinococcus*-tumor of the liver [*3*], Figure 3). He was the first to conclude and publish that the enigmatic emerging disease called alveolar colloid was caused by the larval stage of a previously unknown member of the genus *Echinococcus* and was thus not a form of cancer, as was generally believed. He presented his findings and assumptions during meetings of the scientific society on March 10, 1855, and May 12, 1855.

**Figure 3:**
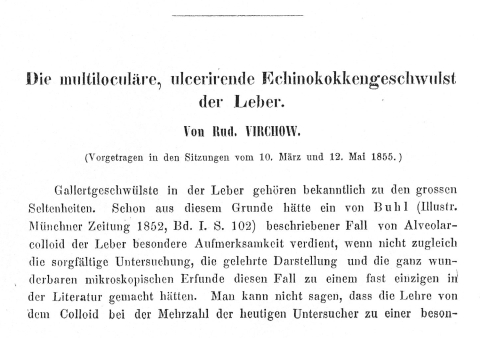
Reproduction of the beginning of Virchow’s original publication (*3*) of a case of hepatic multilocular echinococcosis and his proof that the disease was caused by an *Echinococcus* sp.

## Previous Studies by Buhl and Zeller

Previous to Virchow’s report, Frans Buhl had described the first 2 cases of a strange new condition of the liver, which he called alveolar colloid, in 1852 and 1854, respectively (*4*,*5*). Buhl had found hepatic lesions consisting of many alveoles that contained a gelatinous mass. He believed that the lesions were not a form of a gelatinous cancer (gallertkrebs) as proposed by Meyer (*6*), but rather degenerating tissue (*5*). In 1854, Zeller had reported a similar case (*7*). Both authors provided a detailed description of the new disease, but they were unable to determine its origin or the causative agent.

## Virchow’s Findings

In the introduction of his 1855 article, Virchow pays deference to Buhl and Zeller for the correct description of their histopathologic findings before he describes his own examinations. Virchow states that he is able to add a fourth case to the 3 existing ones. However, he argues that his interpretation of the cases is completely different. Virchow describes how he repeatedly examined the histologic specimens and that the laminated aspect of the gelatinlike membranes and the concentric calcified bodies he observed were in concordance with what Buhl and Zeller had reported about the alveolar colloid. Virchow continues with the statement “already the first glance at the [macroscopic] specimen evoked the imagination of many little echinococcal vesicles.” This opinion seems a bit advanced at this early stage of his investigations, but it nonetheless underscores his interpretation of the alveolar colloid that he would soon rename. After many days of looking at the histologic preparations under the microscope, Virchow was even able to find fully developed protoscolices (third larval stage of the tapeworm) in the vesicles.

From today’s point of view, this finding must be considered a rarity because in human infections with the larval stage of *Echinococcus multilocularis*, hardly any protoscolices are found (*8*). This contrasts with the findings in rodents, the natural intermediate host. This fortunate circumstance guided Virchow to the correct diagnosis, but he stated that he would have come to the same conclusion even without finding any of the “young animals” or their hooklets. In this context, he again mentions observations by Zeller (*7*), who had also found protoscolices in his case of alveolar colloid but had refused to categorize the disease as an echinococcosis. Zeller focused on the differences between the alveolar colloid and the colloidal cancer of the liver. During the time Virchow conducted this study, only the causative agent of hydatid disease, the dog tapeworm *E*. *granulosus* was known. The larval stage of this species develops protoscolices regularly in both humans and its natural intermediate host. The developmental cycle of *E*. *granulosus*, which involves dogs as definitive hosts and sheep as intermediate hosts, was previously elucidated by Carl Theodor Ernst von Siebold in 1853 and 1854 (*9*,*10*).

In his report, Virchow continues with a detailed description of the clinical course of the disease. The patient was a 38-year-old man in whom upper abdominal pain, a short episode of diarrhea, and jaundice developed. The right upper abdomen showed a protrusion, and his physician diagnosed a markedly enlarged liver. The patient was apathetic, his urine was brown, and his stool was the color of sand. Later petechiae, hematemesis, and bloody diarrhea developed. He became rapidly cachectic and died ≈3 months after the onset of jaundice.

Virchow then gives a pictorial description of the autopsy he performed on March 5, 1855. An adherent mass was seen on the surface of the enlarged liver. After sectioning the liver, he observed in the middle of the lesion a large cavum, which was filled with a yellow, puslike fluid. Virchow extensively characterized the cavum, depicting the large lesion as ulcerative. Some peripheral areas of the mass extended into thin rootlike structures, and others formed thick protrusions. The pathologist saw the beginning infiltration of blood vessels by the lesion by which “a further spread would have been possible.” Virchow had described the classic infiltrative aspect and the potential metastasis of lesions of alveolar echinococcosis.

Under the microscope, the lesion consisted of small alveolar vesicles containing gelatin. “The composition of those gelatin-structures, even in concordance with the skin of *echinococcus*, is in many parts so peculiar, that I could hardly resolve my doubts about their nature. Only after some time I was able to find the young animals.” Virchow was about to find protoscolices within the vesicles, “I only found them in the portal parts of the tumor, where the biggest alveoles were present..... They were rounded or of a heart-like shape.…. The hooklets were retracted….. Some of the animals had no hooklets at all and looking at the smaller ones, I would believe that they were still juvenile.” Virchow concluded that the entire tumor consisted of a multitude of exceptionally small echinococcal vesicles, thus the name “multilocular.” He stated that the lesion could therefore no longer be named alveolar colloid. However, he was confused by finding only few protoscolices. Virchow speculated that the protoscolices may have perished in the smaller vesicles or been transformed into a cystic form, which he called acephalocysts. He stated that “there is no longer any doubt about the existence of sterile echinoccocal vesicles in human beings and it seems likely that they developed from immature, hookless [protoscolices].” We know today that in most human patients with alveolar echinococcosis, the parasitic tissue has only sterile vesicles and that protoscolices obtained from lesions of the rodent intermediate host can transform into vesicles in cell culture (*11*). Virchow further concludes that the state of the huge lesion could not be caused by a massive invasion of the liver but must rather be the result of a production of new offspring within the liver. The enormous production of echinococcal vesicles originates from only 1 or a few small oncospheres, which are the invasive larval stage of the parasite.

After Virchow had sent his findings and conclusions to Buhl, Buhl congratulated Virchow for his investigations in a letter (*12*). However, Buhl also wrote that he himself had come to the same conclusions some months before but that he had unfortunately delayed his own publication until it was too late. Buhl stated that he had contacted von Siebold, who had sent him a specimen of a similar echinococcal tumor of an animal liver from his worm collection. This statement led Virchow to conclude that alveolar echinococcosis had a zoonotic aspect. As a pathologist, Virchow read the publications of parasitology and zoology at that time. However, it was believed that the causative organism of alveolar echinococcosis was an aberrant *Echinococcus* sp. and not a separate species. Therefore, other definitive and intermediate hosts than those described by von Siebold for the dog tapeworm were not considered to be involved in the parasite’s life cycle at that time. Buhl’s letter was later presented during a meeting of the scientific society in August 1855 and published in the same volume of the journal as Virchow’s article.

## Further Studies by Others

Soon after Virchow’s article was published, many new cases of this emerging disease were reported (*13*–*17*). Without Virchow’s work, those articles would have described the disease as further cases of alveolar colloid. Thus, the disease had not only been newly recognized but had also been on the increase. In 1863, Leuckart postulated that the newly discovered “multilocular ulcerative *echinococcus*-tumor” is caused by an independent species, which he named *E*. *multilocularis* (*18*). It took another 91 years until the natural cycle involving foxes as definitive hosts and rodents as intermediate hosts was described by Rausch and Schiller in Alaska (*19*) and by Vogel in the Swabian Alb in Germany (*20*). In 1954 and 1955, respectively, it was finally proven that alveolar echinococcosis is caused by the morphologically and biologically distinct species *E*. *multilocularis*.

## Today’s Situation

Since the 1990s, *E. multilocularis* has been spreading, and increased rates of infection in red foxes have been observed in Europe. Today, the range of the parasite extends from central to eastern Europe (*21*). As a consequence, the rate of newly diagnosed alveolar echinococcosis has already doubled in Germany (*22*). In North America, *E*. *multilocularis* is present from Alaska to the Hudson Bay and from southern Canada to the central United States. The parasite will likely spread further since suitable definitive and intermediate hosts are found throughout North America (*23*). Most human cases of alveolar echinococcosis are reported in the People’s Republic of China, where the disease is a serious emerging public health problem (*23*) and a ≤15% prevalence of human alveolar echinococcosis has been reported in some areas (*24*).

More than 150 years after Virchow’s discovery, many questions about alveolar echinococcosis remain unanswered. Why do some persons become infected and exhibit symptoms of this disease, whereas others never show any symptoms of the disease despite being seropositive? It is generally acknowledged that humans contract this disease by ingesting fecally contaminated fruit, such as wood strawberries, in areas where *E*. *multilocularis* is highly prevalent in red foxes. However, the risk factors for acquisition of this disease are still not fully elucidated (*25*). Even today, only a few treatment options exist. Antiparasitic chemotherapy with albendazole or mebendazole has only a parasitostatic effect, and patients have to rely on surgery to cure alveolar echinococcosis, if the disease is diagnosed in time.
